# *CYP11B1* gene polymorphisms and susceptibility to ischemic stroke in a Chinese Han population

**DOI:** 10.3389/fnins.2022.1030551

**Published:** 2022-12-01

**Authors:** Gaowen Liu, Ying Duan

**Affiliations:** Department of Critical Care Medicine, Xianyang Central Hospital, Xianyang, China

**Keywords:** ischemic stroke, susceptibility, *CYP11B1*, gene polymorphisms, case-control study

## Abstract

**Objectives:**

Ischemic stroke (IS) is the major cause of death and disability. While previous studies confirmed that *CYP11B1* is closely associated with IS, the present study aimed to analyze the impact of *CYP11B1* gene polymorphisms on the IS susceptibility.

**Methods:**

The present study genotyped six single nucleotide polymorphisms (SNPs) (including rs4736312, rs5017238, rs5301, rs5283, rs6410, and rs4534) of *CYP11B1* in peripheral blood samples from IS and control populations. Logistic regression analysis was used to analyze the association between the SNPs and IS risk. The multifactor dimensionality reduction (MDR) method was used to determine the roles of SNP–SNP interactions in IS.

**Results:**

The present study showed that rs5283 was associated with an increased susceptibility to IS [odds ratio (OR) 1.81, *p* = 0.012]. On the contrary, rs6410 had a protective influence on IS risk (OR 0.56, *p* = 0.020). Stratified analyses indicated that rs5283 could enhance the risk of IS in subjects aged >63 years (OR 2.41, *p* = 0.011), of female gender (OR 3.31, *p* = 0.001), that do not smoke (OR 1.64, *p* = 0.005), and with hypertension (OR 2.07, *p* = 0.003). Whereas, rs6410 was related to a lower susceptibility to IS in subjects aged >63 years (OR 0.43, *p* = 0.032), of female gender (OR 0.30, *p* = 0.006), do not smoke (OR 0.42, *p* = 0.017), and with hypertension (OR 0.52, *p* = 0.022). Besides, rs4736312 reduced the IS susceptibility in non-smokers (OR 0.69, *p* = 0.031). Rs4534 had a risk-decreasing impact on IS in non-drinking (OR 0.54, *p* = 0.016). Moreover, the results of the MDR analysis corroborate that the best prediction model for IS was rs5283.

**Conclusion:**

This study revealed that *CYP11B1* gene polymorphisms strongly correlated with IS in the Chinese Han population.

## Introduction

Stroke, a common cerebrovascular disease, is the second leading cause of death and the third leading cause of disability and death combined worldwide (Feigin et al., 2021). Ischemic stroke (IS) is the most common type of stroke, accounting for 87% of all stroke cases (Krishnamurthi et al., 2013). In China, stroke is the major cause of death among adults and accounts for a huge burden on medical resources (Wang W. et al., 2017). Stroke-related pain and complications have a phenomenal impact on the patient's quality of life (QoL). At the same time, such complications bring untold misery and burden to the patient's family besides imposing a huge burden on the society at large. Bearing in mind the enormity of treatment to be given to the afflicted patient, effective prevention and therapeutic strategies are warranted and are the need of the hour. Stroke is a multifactorial and complex neurological disorder, including in its fold and scope a host of conventional risk factors, genetic factors, and their interactions. The traditional risk factors for stroke are age, gender, smoking, hypertension, diabetes, obesity, dyslipidemia, etc. (Johnson et al., 2016; Zhang et al., 2019; Zhuo et al., 2020). Previous researches showed that there is a close link between genetic factors and the occurrence of IS (Chauhan and Debette, 2016; Georgakis et al., 2019). Epidemiological studies revealed that gene polymorphisms may be involved in the pathophysiological processes of IS and thereby play a major role in triggering pathophysiological changes occurring in IS (Gao et al., 2019). Moreover, recent studies identified many genetic susceptibility variants which have contributed to the risk of the onset of stroke, such as *ACE* (Goyal et al., 2021), *ADH1B* (Lin et al., 2021), *MTHFR, MTR* (Mialovytska and Nebor, 2021), *IL-10* (Rui et al., 2020), *CYP2J2* (Wang S. Y. et al., 2017), and *CYP2C8* (Yi et al., 2017).

Cytochrome P450 family 11 subfamily B member 1 (*CYP11B1* gene) is located on chromosome 8q24.3 and encodes the steroid 11 β-hydroxylase, which influences the synthesis of aldosterone and activates cellular pathways to promote hypertension and cardiovascular disease (Hussain and Awan, 2018). *CYP11B1* genetic variants are involved in the occurrence and progression of important clinical abnormalities such as late-life depression (Ancelin et al., 2021), Cushing's syndrome (Valassi et al., 2017), hypertensive patients (Hussain et al., 2020), autism (Deng et al., 2016), and coronary heart disease (Huang et al., 2022). The *CYP11B1* and *CYP11B2* genes share 90–95% sequence identity in their non-coding and coding regions. *CYP11B2* gene polymorphisms were found to be associated with the occurrence of IS (Munshi et al., 2010; Yan and Wang, 2012). Taking all these facts together, we speculated that *CYP11B1* gene polymorphisms may have an important role in the development of IS. However, the role of *CYP11B1* gene polymorphism in IS has not yet been reported.

In this study, we carried out a case-control study (that included 550 IS patients and 550 normal populations) to explore the correlation between *CYP11B1* gene polymorphisms and IS susceptibility in the Chinese Han population. Our study provides a new biomarker for the prevention and diagnosis of IS.

## Materials and methods

The present study was approved by the Ethics Committee of the Xianyang Central Hospital. We explained to each participant the purpose of undertaking this study and also obtained an informed consent from the concerned participants before the commencement of the study. In this study, we randomly recruited 550 patients with IS and age- and sex-matched 550 healthy populations from the Xianyang Central Hospital during the same period. The inclusion criteria for the study required only those patients who were newly diagnosed as having IS and who should have been confirmed to have suffered IS by two experienced neurologists based on the test records of clinical examination, magnetic resonance imaging (MRI), cerebral scanner, and/ or computed tomography (CT) in accordance with the diagnostic guidelines for stroke (Liberman et al., 2016). Patients who possessed any of the exclusion criteria were ruled out from participation. The exclusion criteria for the cases were as follows: (1) patients with genetic disease; (2) patients with a family history of stroke; (3) patients with any type of cancer, including brain tumor; and (4) patients with neurological, cardiogenic, and autoimmune diseases. The control group included a healthy population who had undergone physical examination. The inclusion criteria stipulated for the controls were as follows: (1) controls matched to cases for age and gender and (2) controls with no family history of brain and neurological diseases. The basic characteristics of all participants were fetched from medical records and a standardized demographic questionnaire that included the participant's health details such as age, gender, smoking status, alcohol intake, hypertension status, total cholesterol, triglycerides, high-density lipoprotein cholesterol (HDL-c), and low-density lipoprotein cholesterol (LDL-c).

### SNP selection and genotyping

The detailed steps that had to be undertaken for the selection of *CYP11B1* single nucleotide polymorphisms (SNPs) were as follows: (1) We obtained the physical position of the *CYP11B1* gene on the chromosome 8:142872356-142879846 through the human Ensembl GRCh37 database (http://asia.ensembl.org/Homo_sapiens/Info/Index). In the VCF to PED Converter window (http://grch37.ensembl.org/Homo_sapiens/Tools/VcftoPed), we entered the gene location, selected the Chinese Han population in Beijing (CHB) population, and downloaded the PED and info file for the SNPs of *CYP11B1*. We obtained 31 SNPs within *CYP11B1* from the database. (2) Then, we used Haploview software for quality control [minor allele frequency (MAF) > 5%, min genotype > 75%, *r*^2^ <0.8, and Hardy–Weinberg equilibrium (HWE) > 0.05] to select the tag-SNP. (3) The call rate of each SNP was >95%. Other SNPs in the *CYP11B1* gene did not meet the above standards. Finally, six SNPs (including rs4736312, rs5017238, rs5301, rs5283, rs6410, and rs4534) that met the above standards were selected for investigation and further study. Genomic DNA from the peripheral blood samples was extracted using the DNA kit. The primers for polymerase chain reaction (PCR) amplification were designed by the Agena Design software. The six SNPs were detected by the Agena MassARRAY iPLEX platform following the manufacturer's protocols. In addition, the genotyping data were analyzed by the Agena Bioscience Typer software.

### Bioinformatics analysis

Haploreg (Version 4.1) online software was used to predict the possible functions of the six SNPs(https://pubs.broadinstitute.org/mammals/haploreg/haploreg.php).

### Statistical analyses

In this study, statistical tests were analyzed by SPSS software (version 22.0), with a two-tailed test. Student's *t*-test was performed to detect the statistical differences in age, total cholesterol, triglycerides, HDL-c, and LDL-c between the case and control groups, respectively. Pearson's chi-squared (χ^2^) test was used to analyze the statistical differences in gender, smoking status, and alcohol intake. Fisher's exact test was carried out to calculate the HWE to detect the allele frequencies in normal controls. The association of *CYP11B1* gene polymorphisms with IS susceptibility was evaluated by a logistic regression analysis under allele, codominant, dominant, recessive, and log-additive models. The Benjamini and Hochberg's false discovery rate (FDR) method was used to correct for multiple comparisons. Besides, the positive findings about the correlations between SNPs and IS risk were verified with the false-positive report probability (FPRP) analysis. Moreover, the MDR method was used to determine the influence of interactions among SNPs on IS susceptibility.

## Results

### Basic characteristics of the study population

As shown in Table 1, our study involved 550 patients with IS (341 men and 209 women) and 550 healthy subjects (326 men and 224 women). The mean age was 63.01 ± 7.44 years for the cases and 63.71 ± 10.53 years for the controls. The concentrations of total cholesterol, HDL-c, and LDL-c in patients with IS were significantly lower than those in the control group (all *p* < 0.001). In terms of age, gender, smoking status, alcohol intake, and triglyceride level, there was no significant difference between the two groups (*p* = 0.203, *p* = 0.355, *p* = 0.469, *p* = 0.763, and *p* = 0.114, respectively).

**Table 1:** Basic characteristics of participants in this study.

**Variables**	**Cases (*n* = 550)**	**Controls (*n* = 550)**	***P***
Age, years (mean ± SD)^a^	63.01 ± 7.44	63.71 ± 10.53	0.203
>63	275 (50%)	270 (49.1%)	
≤ 63	275 (50%)	280 (50.9%)	
Gender^b^			0.355
Male	341 (62%)	326 (59.3%)	
Female	209 (38%)	224 (40.7%)	
Smoking status^b^			0.469
Smoker	283 (51.5%)	271 (49.3%)	
Non-smoker	267 (48.5%)	279 (50.7%)	
Alcohol intake^b^			0.763
Yes	284 (51.6%)	279 (50.7%)	
No	266 (48.4%)	271 (49.3%)	
Hypertension status			
No	165 (30%)		
Yes	385 (70%)		
Total cholesterol (mmol/l)^a^	3.93 ± 0.87	4.74 ± 0.89	**<0.001**
Triglycerides (mmol/l)^a^	1.55 ± 0.92	1.62 ± 0.68	0.114
HDL-c (mmol/l)^a^	1.16 ± 0.26	1.25 ± 0.26	**<0.001**
LDL-c (mmol/l)^a^	2.05 ± 0.63	2.58 ± 0.62	**<0.001**

HDL-C, high-density lipoprotein cholesterol; LDL-C, low-density lipoprotein cholesterol.

a Student's t-test is used.

b Pearson's X^2^ test is used.

*p* < 0.05 indicates statistical significance.

Significant *p*-values are given in bold.

### The impact of *CYP11B1* gene polymorphisms on ischemic stroke susceptibility

We successfully investigated six SNPs (rs4736312, rs5017238, rs5301, rs5283, rs6410, and rs4534), and the allele frequency distribution and the potential function of the SNPs are listed in Table 2. The allele frequencies for each SNP in the controls were assigned in accordance with the HWE (all *p* > 0.05). The association of *CYP11B1* polymorphisms with IS is listed in Table 3. Rs5283 was significantly associated with an increased susceptibility to IS in allele [OR 1.32, *p* = 0.003, *p* (FDR) = 0.020], codominant [GA vs. GG, OR 1.33, *p* = 0.026, *p* (FDR) = 0.153; AA vs. GG, OR 1.81, *p* = 0.012, *p* (FDR) = 0.072], dominant [OR 1.39, *p* = 0.007, *p* (FDR) = 0.040], recessive [OR 1.58, *p* = 0.044, *p* (FDR) = 0.133], and log-additive models [OR 1.34, *p* = 0.003, *p* (FDR) = 0.016]. Whereas, rs6410 had a risk-decreasing influence on IS in allele [OR 0.81, *p* = 0.027, *p* (FDR) = 0.080], codominant [TT vs. CC, OR 0.56, *p* = 0.020, *p* (FDR) = 0.061], recessive [OR 0.60, *p* = 0.037, *p* (FDR) = 0.224], and log-additive models [OR 0.80, *p* = 0.022, *p* (FDR) = 0.065].

**Table 2:** The distribution of allele frequencies of *CYP11B1* SNPs.

**SNP ID**	**Chromosome position**	**Role**	**Alleles (A/B)**	**Callrate**	**MAF**		***p*-HWE**	**HaploReg v4.1**
					**Case**	**Control**		
rs4736312	chr8: 142872521	3′-UTR	A/C	99.9%	0.154	0.170	0.652	DNAse, Motifs changed, Selected eQTL hits
rs5017238	chr8: 142873353	3′-UTR	G/A	99.9%	0.158	0.171	1.000	Promoter histone marks, DNAse, Motifs changed, Selected eQTL hits
rs5301	chr8: 142873857	3′-UTR	T/C	100%	0.160	0.171	0.652	Promoter histone marks, Motifs changed, GRASP QTL hits, Selected eQTL hits
rs5283	chr8: 142879181	Synonymous	A/G	99.7%	0.326	0.269	0.385	DNAse, Motifs changed, Selected eQTL hits
rs6410	chr8: 142879589	Synonymous	T/C	100%	0.265	0.308	0.319	Promoter histone marks, Enhancer histone marks, Motifs changed, GRASP QTL hits, Selected eQTL hits
rs4534	chr8: 142879686	Missense	T/C	99.9%	0.400	0.419	1.000	Promoter histone marks, Enhancer histone marks, DNAse, Motifs changed

SNPs, single nucleotide polymorphisms; A, minor allele; B, major allele; MAF, minor allele frequency; HWE, Hardy–Weinberg equilibrium.

*p* < 0.05 are excluded.

**Table 3:** Association between *CYP11B1* gene polymorphisms and ischemic stroke susceptibility.

**SNP ID**	**Model**	**Allele/Genotype**	**Case N**	**Control N**	**OR (95% CI)**	***p***	***p* (FDR)**
rs4736312	Allele	C	931	911	1		
		A	169	187	0.88 (0.70–1.11)	0.289	0.577
	Codominant	AC	153	159	0.92 (0.71–1.20)	0.530	1.060
		AA	8	14	0.55 (0.23–1.34)	0.191	0.382
		CC	389	376	1		
	Dominant	AC-AA	161	173	0.89 (0.69–1.15)	0.373	0.747
	Recessive	CC-AC	542	535	1		
		AA	8	14	0.57 (0.24–1.37)	0.208	0.312
	Log-additive	–	–	–	0.87 (0.69–1.10)	0.248	0.497
rs5017238	Allele	A	925	912	1		
		G	173	188	0.91 (0.72–1.14)	0.398	0.478
	Codominant	AG	149	156	0.92 (0.70–1.20)	0.536	0.804
		GG	12	16	0.73 (0.34–1.56)	0.417	0.417
		AA	388	378	1		
	Dominant	AG-GG	161	172	0.90 (0.70–1.17)	0.432	0.648
	Recessive	AA-AG	537	534	1		
		GG	12	16	0.75 (0.35–1.60)	0.451	0.451
	Log-additive	–	–	–	0.90 (0.72–1.13)	0.357	0.428
rs5301	Allele	C	924	912	1		
		T	176	188	0.92 (0.74–1.16)	0.491	0.491
	Codominant	TC	158	160	0.96 (0.74–1.25)	0.747	0.747
		TT	9	14	0.63 (0.27–1.48)	0.289	0.346
		CC	383	376	1		
	Dominant	TC-TT	167	174	0.93 (0.72–1.20)	0.587	0.704
	Recessive	CC-TC	541	536	1		
		TT	9	14	0.64 (0.27–1.49)	0.300	0.360
	Log-additive	–	–	–	0.91 (0.72–1.15)	0.435	0.435
rs5283	Allele	G	740	803	1		
		A	358	295	1.32 (1.10–1.58)	**0.003**	**0.020**
	Codominant	GA	252	225	1.33 (1.04–1.70)	**0.026**	0.153
		AA	53	35	1.81 (1.14–2.86)	**0.012**	0.072
		GG	244	289	1		
	Dominant	GA-AA	305	260	1.39 (1.10–1.77)	**0.007**	**0.040**
	Recessive	GG-GA	496	514	1		
		AA	53	35	1.58 (1.01–2.47)	**0.044**	0.133
	Log-additive	–	–	–	1.34 (1.11–1.61)	**0.003**	**0.016**
rs6410	Allele	C	808	761	1		
		T	292	339	0.81 (0.67–0.98)	**0.027**	0.080
	Codominant	TC	234	245	0.85 (0.67–1.09)	0.207	0.620
		TT	29	47	0.56 (0.34–0.91)	**0.020**	0.061
		CC	287	258	1		
	Dominant	TC-TT	263	292	0.81 (0.64–1.02)	0.075	0.224
	Recessive	CC-TC	521	503	1		
		TT	29	47	0.60 (0.37–0.97)	**0.037**	0.224
	Log-additive	–	–	–	0.80 (0.66–0.97)	**0.022**	0.065
rs4534	Allele	C	660	639	1		
		T	440	461	0.92 (0.78–1.10)	0.363	0.544
	Codominant	TC	288	269	1.06 (0.81–1.37)	0.683	0.820
		TT	76	96	0.79 (0.55–1.14)	0.206	0.308
		CC	186	185	1		
	Dominant	TC-TT	364	365	0.99 (0.77–1.27)	0.916	0.916
	Recessive	CC-TC	474	454	1		
		TT	76	96	0.76 (0.55–1.06)	0.109	0.219
	Log-additive	–	–	–	0.92 (0.77–1.10)	0.352	0.528

SNP: single nucleotide polymorphism; OR, odds ratio, 95% CI; 95% confidence intervals; FDR, false discovery rate.

p-values were calculated by unconditional logistic regression analysis with adjustment for age, gender, smoking, and drinking.

A *p*-value of < 0.05 indicates statistical significance.

Significant *p*-values are given in bold.

### *CYP11B1* SNPs associated with risk factors for ischemic stroke

We further analyzed the impact of *CYP11B1* SNPs on risk factors (gender, age, alcohol intake, smoking, and hypertension) for patients with IS. As shown in Table 4, age-stratified analysis indicated that rs5283 was related to an increased risk of IS in people aged >63 years [allele: OR 1.38, *p* = 0.015, *p* (FDR) = 0.089; codominant: GA vs. GG, OR 1.58, *p* = 0.018, *p* (FDR) = 0.108, and AA vs. GG, OR 2.41, *p* = 0.011, *p* (FDR) = 0.032; dominant: OR 1.69, *p* = 0.005, *p* (FDR) = 0.028; recessive: OR 1.93, *p* = 0.047, *p* (FDR) = 0.282; log-additive: OR 1.56, *p* = 0.002, *p* (FDR) = 0.013]. Rs6410 was related to a lower risk in ischemic stroke aged >63 years under codominant [TT vs. CC, OR 0.43, *p* = 0.032, *p* (FDR) = 0.194] and log-additive models [OR 0.72, *p* = 0.031, *p* (FDR) = 0.094]. When stratified by gender, it was found that rs5283 showed an enhanced susceptibility to IS in women [allele: OR 1.87, *p* < 0.001, *p* (FDR) < 0.001; codominant: GA vs. GG, OR 2.24, *p* < 0.001, *p* (FDR) = 0.001, and AA vs. GG, OR 3.31, *p* = 0.001, *p* (FDR) = 0.009; dominant: OR 2.39, *p* < 0.001, *p* (FDR) <0.001; recessive: OR 2.25, *p* = 0.024, *p* (FDR) = 0.073; log-additive: OR 1.99, *p* < 0.001, *p* (FDR) < 0.001]. However, rs6410 [allele: OR 0.64, *p* = 0.003, *p* (FDR) = 0.009; codominant: TC vs. CC, OR 0.67, *p* = 0.045, *p* (FDR) = 0.136, and TT vs. CC, OR 0.30, *p* = 0.006, *p* (FDR) = 0.017; dominant: OR 0.60, *p* = 0.009, *p* (FDR) = 0.028; recessive: OR 0.36, *p* = 0.017, *p* (FDR) = 0.103; log-additive: OR 0.61, *p* = 0.002, *p* (FDR) = 0.006] and rs4534 [recessive: OR 0.58, *p* = 0.046, *p* (FDR) = 0.093] had a protective impact on the risk of IS in women.

**Table 4:** Correlation of *CYP11B1* polymorphisms and ischemic stroke susceptibility stratified by age and gender.

**SNP ID**	**Model**	**Allele/ Genotype**	>**63 years**			≤ **63 years**			**Men**			**Women**		
			**OR (95% CI)**	***p^*a*^***	***p* (FDR)**	**OR (95% CI)**	***p^*a*^***	***p* (FDR)**	**OR (95% CI)**	***p^*b*^***	***p* (FDR)**	**OR (95% CI)**	***p^*b*^***	***p* (FDR)**
rs4736312	Allele	C	1			1			1			1		
		A	0.86 (0.62–1.18)	0.345	0.517	0.91 (0.6–1.26)	0.575	1.725	0.85 (0.64–1.13)	0.260	1.558	0.94 (0.65–1.36)	0.746	1.119
	Codominant	AC	0.82 (0.56–1.22)	0.334	0.668	0.99 (0.67–1.46)	0.946	1.135	0.93 (0.66–1.30)	0.653	1.305	0.89 (0.57–1.37)	0.583	0.874
		AA	0.38 (0.08–1.67)	0.198	0.297	0.63 (0.20–2.00)	0.428	0.856	0.40 (0.12–1.31)	0.131	0.785	1.03 (0.25–4.23)	0.968	0.968
		CC	1			1			1			1		
	Dominant	AC-AA	0.79 (0.54–1.16)	0.234	0.467	0.95 (0.65–1.39)	0.796	1.591	0.88 (0.63–1.22)	0.439	2.636	0.89 (0.59–1.37)	0.605	0.907
	Recessive	CC-AC	1			1			1			1		
		AA	0.40 (0.09–1.75)	0.223	0.335	0.63 (0.20–1.99)	0.430	0.860	0.41 (0.13–1.34)	0.139	0.835	1.06 (0.26–4.35)	0.932	1.118
	Log-additive	–	0.78 (0.54–1.11)	0.159	0.239	0.92 (0.66–1.29)	0.641	1.282	0.84 (0.63–1.14)	0.263	1.576	0.92 (0.63–1.34)	0.656	0.984
rs5017238	Allele	A	1			1			1			1		
		G	0.89 (0.65–1.22)	0.451	0.541	0.93 (0.67–1.28)	0.655	1.310	0.86 (0.65–1.15)	0.316	0.947	0.97 (0.67–1.41)	0.893	0.893
	Codominant	AG	0.83 (0.56–1.24)	0.364	0.546	0.98 (0.66–1.46)	0.931	1.396	0.92 (0.66–1.30)	0.648	1.943	0.88 (0.57–1.37)	0.578	1.155
		GG	0.55 (0.16–1.91)	0.344	0.413	0.75 (0.27–2.11)	0.590	0.708	0.57 (0.22–1.48)	0.248	0.496	1.36 (0.35–5.21)	0.655	0.983
		AA	1			1			1			1		
	Dominant	AG-GG	0.81 (0.55–1.19)	0.282	0.422	0.96 (0.65–1.40)	0.819	1.229	0.88 (0.64–1.23)	0.467	1.400	0.91 (0.60–1.39)	0.671	0.671
	Recessive	AA-AG	1			1			1			1		
		GG	0.58 (0.17–2.00)	0.388	0.388	0.76 (0.27–2.11)	0.594	0.713	0.58 (0.22–1.51)	0.265	0.531	1.40 (0.37–5.36)	0.621	0.931
	Log-additive	–	0.81 (0.57–1.14)	0.225	0.270	0.94 (0.68–1.31)	0.712	0.854	0.86 (0.65–1.15)	0.323	0.970	0.96 (0.65–1.40)	0.812	0.812
rs5301	Allele	C	1			1			1			1		
		T	0.92 (0.67–1.26)	0.590	0.590	0.93 (0.67–1.28)	0.658	0.986	0.91 (0.68–1.21)	0.506	1.012	0.94 (0.65–1.36)	0.752	0.903
	Codominant	TC	0.87 (0.59–1.28)	0.473	0.568	1.01 (0.68–1.48)	0.976	0.976	0.99 (0.70–1.38)	0.937	1.406	0.89 (0.58–1.37)	0.608	0.729
		TT	0.52 (0.14–2.02)	0.346	0.346	0.63 (0.20–2.02)	0.440	0.660	0.51 (0.17–1.52)	0.223	0.669	1.03 (0.25–4.24)	0.966	1.159
		CC	1			1			1			1		
	Dominant	TC-TT	0.84 (0.57–1.23)	0.377	0.452	0.97 (0.67–1.41)	0.875	0.875	0.94 (0.68–1.31)	0.725	1.450	0.90 (0.59–1.37)	0.629	0.755
	Recessive	CC-TC	1			1			1			1		
		TT	0.55 (0.14–2.09)	0.377	0.452	0.63 (0.20–2.01)	0.436	0.654	0.51 (0.17–1.51)	0.224	0.672	1.06 (0.26–4.35)	0.932	1.118
	Log-additive	–	0.83 (0.59–1.18)	0.298	0.298	0.94 (0.67–1.31)	0.710	1.065	0.90 (0.67–1.21)	0.504	1.008	0.92 (0.63–1.35)	0.679	0.814
rs5283	Allele	G	1			1			1			1		
		A	1.38 (1.07–1.79)	**0.015**	0.089	1.26 (0.97–1.63)	1.000	1.000	1.05 (0.83–1.33)	0.692	0.831	1.87 (1.40–2.52)	**<0.001**	**<0.001**
	Codominant	GA	1.58 (1.08–2.30)	**0.018**	0.108	1.22 (0.86–1.74)	0.271	0.814	1.01 (0.73–1.39)	0.955	1.146	2.24 (1.48–3.37)	**<0.001**	**0.001**
		AA	2.41 (1.23–4.73)	**0.011**	**0.032**	2.00 (1.00–3.99)	0.051	0.303	1.22 (0.66–2.23)	0.527	0.632	3.31 (1.58–6.90)	**0.001**	**0.009**
		GG	1			1			1			1		
	Dominant	GA-AA	1.69 (1.18–2.42)	**0.005**	**0.028**	1.31 (0.93–1.84)	0.123	0.739	1.04 (0.76–1.41)	0.818	1.227	2.39 (1.61–3.53)	**<0.001**	**<0.001**
	Recessive	GG-GA	1			1			1			1		
		AA	1.93 (1.01–3.68)	**0.047**	0.282	1.82 (0.93–3.56)	0.082	0.489	1.21 (0.67–2.18)	0.523	0.784	2.25 (1.11–4.56)	**0.024**	0.073
	Log-additive	–	1.56 (1.18–2.08)	**0.002**	**0.013**	1.32 (1.00–1.74)	0.050	0.297	1.06 (0.83–1.35)	0.651	0.976	1.99 (1.46–2.72)	**<0.001**	**<0.001**
rs6410	Allele	C	1			1			1			1		
		T	0.81 (0.63–1.06)	0.126	0.378	0.81 (0.62–1.05)	0.109	0.652	0.95 (0.75–1.20)	0.645	0.967	0.64 (0.47–0.86)	**0.003**	**0.009**
	Codominant	TC	0.79 (0.55–1.15)	0.218	0.655	0.80 (0.56–1.15)	0.224	1.344	1.00 (0.73–1.37)	0.986	0.986	0.67 (0.45–0.99)	**0.045**	0.136
		TT	0.43 (0.20–0.93)	**0.032**	0.194	0.59 (0.30–1.16)	0.123	0.369	0.83 (0.44–1.54)	0.550	0.824	0.30 (0.13–0.70)	**0.006**	**0.017**
		CC	1			1			1			1		
	Dominant	TC-TT	0.73 (0.51–1.05)	0.092	0.277	0.76 (0.54–1.08)	0.124	0.372	0.97 (0.72–1.32)	0.855	0.855	0.60 (0.41–0.88)	**0.009**	**0.028**
	Recessive	CC-TC	1			1			1			1		
		TT	0.48 (0.23–1.02)	0.056	0.169	0.65 (0.34–1.25)	0.198	0.593	0.83 (0.45–1.52)	0.541	0.541	0.36 (0.16–0.83)	**0.017**	0.103
	Log-additive	–	0.72 (0.54–0.97)	**0.031**	0.094	0.78 (0.60–1.03)	0.078	0.234	0.95 (0.74–1.22)	0.690	0.828	0.61 (0.44–0.83)	**0.002**	**0.006**
rs4534	Allele	C	1			1			1			1		
		T	0.85 (0.67–1.09)	0.194	0.388	1.00 (0.79–1.27)	0.993	1.192	0.99 (0.80–1.23)	0.940	0.940	0.83 (0.63–1.09)	0.170	0.341
	Codominant	TC	0.96 (0.65–1.42)	0.835	0.835	1.07 (0.73–1.57)	0.714	1.428	1.07 (0.76–1.51)	0.684	4.106	1.01 (0.66–1.54)	0.963	0.963
		TT	0.62 (0.35–1.10)	0.103	0.206	0.95 (0.58–1.58)	0.857	0.857	0.91 (0.57–1.47)	0.708	0.708	0.59 (0.33–1.05)	0.074	0.148
		CC	1			1			1			1		
	Dominant	TC-TT	0.87 (0.60–1.28)	0.487	0.487	1.04 (0.73–1.49)	0.824	0.989	1.03 (0.75–1.43)	0.839	1.006	0.89 (0.59–1.32)	0.552	1.103
	Recessive	CC-TC	1			1			1			1		
		TT	0.64 (0.38–1.07)	0.088	0.177	0.92 (0.58–1.44)	0.703	0.703	0.87 (0.57–1.34)	0.537	0.645	0.58 (0.34–0.99)	**0.046**	0.093
	Log-additive	–	0.83 (0.63–1.08)	0.163	0.326	0.99 (0.78–1.27)	0.956	0.956	0.98 (0.78–1.23)	0.850	0.850	0.81 (0.61–1.07)	0.139	0.278

OR, odds ratio, 95% CI; 95% confidence intervals; FDR, false discovery rate.

The *p*^a^ values were calculated by logistic regression analysis adjusted by gender, smoking, and drinking. The *p*^b^ values were calculated by logistic regression analysis adjusted by age, smoking, and drinking.

A *p*-value of < 0.05 indicates statistical significance.

Significant *p*-values are given in bold.

After stratified by smoking and alcohol intake (Table 5), we found that rs4736312 [allele: OR 0.72, *p* = 0.045, *p* (FDR) = 0.091; log-additive: OR 0.69, *p* = 0.031, *p* (FDR) = 0.063] and rs6410 [OR 0.74, *p* = 0.027, *p* (FDR) = 0.082; codominant: OR 0.42, *p* = 0.017, *p* (FDR) = 0.104; recessive: OR 0.46, *p* = 0.029, *p* (FDR) = 0.172; log-additive: OR 0.73, *p* = 0.024, *p* (FDR) = 0.073] could decrease the risk of patients that do not smoke. In addition, rs5283 [allele: OR 1.40, *p* = 0.012, *p* (FDR) = 0.070; codominant: GA vs. GG, OR 1.62, *p* = 0.008, *p* (FDR) = 0.051; dominant: OR 1.64, *p* = 0.005, *p* (FDR) = 0.029; log-additive: OR 1.45, *p* = 0.008, *p* (FDR) = 0.047] could increase the susceptibility of IS in patients that do not smoke. Rs4534 was related to decreased susceptibility in patients that do not consume alcohol under codominant [TT vs. CC, OR 0.57, *p* = 0.041, *p* (FDR) = 0.245] and recessive models [OR 0.54, *p* = 0.016, *p* (FDR) = 1.977]. We further evaluated the correlations between SNPs and IS complicated with hypertension. As summarized in Table 6, rs5283 significantly increased the risk of IS complicated with hypertension in allele [OR 1.34, *p* = 0.004, *p* (FDR) = 0.026], codominant [AA vs. GG, OR 2.07, *p* = 0.003, *p* (FDR) = 0.020], dominant [OR 1.35, *p* = 0.024, *p* (FDR) = 0.143], recessive [OR 1.87, *p* = 0.009, *p* (FDR) = 0.233], and log-additive models [OR 1.35, *p* = 0.004, *p* (FDR) = 0.022]. Rs6410 had a lower susceptibility to IS complicated with hypertension [allele: OR 0.80, *p* = 0.031, *p* (FDR) = 0.094, codominant: OR 0.52, *p* = 0.022, *p* (FDR) = 0.067, recessive: OR 0.56, *p* = 0.039, *p* (FDR) = 0.875, and log-additive: OR 0.78, *p* = 0.025, *p* (FDR) = 0.074].

**Table 5:** Correlation of *CYP11B1* polymorphisms and ischemic stroke susceptibility stratified by smoking and drinking.

**SNP ID**	**Model**	**Allele/Genotype**	**Smoking**			**Non-smoking**			**Drinking**			**Non-drinking**		
			**OR (95% CI)**	***p^*a*^***	***p* (FDR)**	**OR (95% CI)**	***p^*a*^***	***p* (FDR)**	**OR (95% CI)**	***p^*b*^***	***p* (FDR)**	**OR (95% CI)**	***p^*b*^***	***p* (FDR)**
rs4736312	Allele	C	1			1			1			1		
		A	1.09 (0.79–1.50)	0.615	0.615	0.72 (0.52–0.99)	**0.045**	0.091	0.95 (0.70–1.30)	0.769	1.538	0.81 (0.58–1.13)	0.207	0.311
	Codominant	AC	1.05 (0.72–1.52)	0.813	0.813	0.74 (0.50–1.09)	0.128	0.257	1.02 (0.70–1.47)	0.934	1.120	0.75 (0.51–1.12)	0.163	0.326
		AA	1.40 (0.29–6.67)	0.672	0.672	0.36 (0.11–1.16)	0.086	0.258	0.24 (0.05–1.22)	0.085	0.254	0.88 (0.29–2.71)	0.829	0.994
		CC	1			1			1			1		
	Dominant	AC-AA	1.06 (0.73–1.53)	0.763	0.763	0.70 (0.48–1.01)	0.059	0.176	0.96 (0.67–1.37)	0.802	2.406	0.76 (0.52–1.12)	0.170	0.511
	Recessive	CC-AC	1			1			1			1		
		AA	1.38 (0.29–6.56)	0.684	0.820	0.39 (0.12–1.25)	0.113	0.339	0.24 (0.05–1.20)	0.083	0.248	0.95 (0.31–2.90)	0.928	0.820
	Log-additive	–	1.07 (0.76–1.51)	0.708	0.708	0.69 (0.50–0.97)	**0.031**	0.063	0.89 (0.64–1.24)	0.490	0.980	0.81 (0.58–1.13)	0.218	0.327
rs5017238	Allele	A	1			1			1			1		
		G	1.11 (0.81–1.53)	0.513	0.616	0.74 (0.53–1.02)	0.063	0.076	0.97 (0.71–1.32)	0.829	1.244	0.84 (0.60–1.17)	0.302	0.362
	Codominant	AG	1.05 (0.72–1.54)	0.796	1.195	0.73 (0.50–1.08)	0.120	0.359	1.03 (0.71–1.50)	0.863	1.725	0.74 (0.49–1.10)	0.138	0.415
		GG	1.41 (0.43–4.65)	0.569	0.854	0.46 (0.16–1.37)	0.165	0.198	0.40 (0.12–1.36)	0.144	0.287	1.15 (0.40–3.27)	0.793	1.190
		AA	1			1			1			1		
	Dominant	AG-GG	1.07 (0.74–1.56)	0.708	1.062	0.70 (0.48–1.02)	0.066	0.132	0.97 (0.68–1.39)	0.870	1.305	0.77 (0.53–1.14)	0.189	0.378
	Recessive	AA-AG	1			1			1			1		
		GG	1.39 (0.43–4.56)	0.584	0.876	0.50 (0.17–1.49)	0.214	0.320	0.40 (0.12–1.34)	0.137	0.274	1.24 (0.44–3.51)	0.683	1.403
	Log-additive	–	1.09 (0.78–1.52)	0.623	0.747	0.72 (0.51–1.00)	0.047	0.070	0.91 (0.66–1.26)	0.558	0.837	0.84 (0.60–1.18)	0.315	0.378
rs5301	Allele	C	1			1			1			1		
		T	1.16 (0.84–1.59)	0.372	0.558	0.74 (0.53–1.02)	0.061	0.091	0.98 (0.72–1.33)	0.891	1.069	0.86 (0.62–1.20)	0.381	0.381
	Codominant	TC	1.10 (0.76–1.60)	0.609	1.217	0.77 (0.52–1.13)	0.184	0.276	1.02 (0.71–1.48)	0.901	1.352	0.83 (0.56–1.22)	0.336	0.504
		TT	1.75 (0.39–7.75)	0.462	0.923	0.36 (0.11–1.17)	0.090	0.135	0.37 (0.09–1.49)	0.161	0.241	0.91 (0.30–2.78)	0.864	0.864
		CC	1			1			1			1		
	Dominant	TC-TT	1.13 (0.78–1.63)	0.530	1.060	0.72 (0.50–1.05)	0.089	0.134	0.97 (0.68–1.39)	0.877	1.052	0.83 (0.57–1.22)	0.341	0.409
	Recessive	CC-TC	1			1			1			1		
		TT	1.70 (0.38–7.49)	0.485	0.970	0.39 (0.12–1.26)	0.114	0.229	0.37 (0.09–1.47)	0.156	**0.031**	0.95 (0.31–2.91)	0.935	0.540
	Log-additive	–	1.14 (0.81–1.61)	0.452	0.678	0.72 (0.51–1.00)	0.047	0.056	0.92 (0.66–1.28)	0.603	0.723	0.86 (0.62–1.21)	0.388	0.388
rs5283	Allele	G	1			1			1			1		
		A	1.25 (0.96–1.62)	0.098	0.586	1.40 (1.08–1.81)	**0.012**	0.070	1.15 (0.89–1.49)	0.286	0.859	1.51 (1.17–1.96)	**0.002**	**0.011**
	Codominant	GA	1.14 (0.80–1.63)	0.460	1.379	1.62 (1.13–2.32)	**0.008**	0.051	0.91 (0.64–1.30)	0.617	1.852	2.00 (1.39–2.89)	**<0.001**	**0.001**
		AA	1.90 (0.95–3.82)	0.071	0.424	1.76 (0.92–3.34)	0.086	0.172	2.20 (1.07–4.52)	**0.032**	0.190	1.69 (0.90–3.19)	0.102	0.204
		GG	1			1			1			1		
	Dominant	GA-AA	1.23 (0.87–1.73)	0.238	1.426	1.64 (1.16–2.32)	**0.005**	**0.029**	1.03 (0.74–1.44)	0.862	1.723	1.95 (1.37–2.76)	**<0.001**	**0.001**
	Recessive	GG-GA	1			1			1			1		
		AA	1.79 (0.91–3.51)	0.093	0.280	1.39 (0.75–2.58)	0.296	0.355	2.29 (1.14–4.62)	**0.020**	1.413	1.21 (0.66–2.21)	0.540	0.179
	Log-additive	–	1.26 (0.96–1.66)	0.099	0.593	1.45 (1.10–1.90)	**0.008**	**0.047**	1.17 (0.89–1.53)	0.266	0.798	1.55 (1.18–2.04)	**0.002**	**0.010**
rs6410	Allele	C	1			1			1			1		
		T	0.88 (0.68–1.15)	0.357	0.714	0.74 (0.57–0.97)	**0.027**	0.082	0.84 (0.65–1.08)	0.171	1.024	0.78 (0.60–1.03)	0.075	0.225
	Codominant	TC	0.88 (0.62–1.26)	0.482	2.891	0.82 (0.58–1.17)	0.281	0.337	0.84 (0.59–1.20)	0.336	2.014	0.87 (0.61–1.24)	0.441	0.530
		TT	0.83 (0.41–1.72)	0.622	0.747	0.42 (0.21–0.86)	**0.017**	0.104	0.61 (0.31–1.23)	0.166	0.200	0.51 (0.25–1.05)	0.068	0.203
		CC	1			1			1			1		
	Dominant	TC-TT	0.87 (0.62–1.23)	0.443	1.329	0.75 (0.53–1.05)	0.096	0.115	0.81 (0.58–1.13)	0.214	1.283	0.81 (0.57–1.14)	0.224	0.336
	Recessive	CC-TC	1			1			1			1		
		TT	0.89 (0.44–1.79)	0.735	0.735	0.46 (0.23–0.92)	**0.029**	0.172	0.67 (0.34–1.31)	0.236	1.080	0.54 (0.27–1.10)	0.090	**0.048**
	Log-additive	–	0.90 (0.68–1.19)	0.445	0.890	0.73 (0.55–0.96)	**0.024**	0.073	0.81 (0.62–1.07)	0.135	0.811	0.79 (0.60–1.04)	0.093	0.279
rs4534	Allele	C	1			1			1			1		
		T	0.85 (0.69–1.12)	0.299	0.896	0.97 (0.76–1.23)	0.785	0.785	1.01 (0.79–1.28)	0.960	0.960	0.84 (0.66–1.08)	0.171	0.343
	Codominant	TC	1.05 (0.71–1.54)	0.807	0.968	1.02 (0.70–1.48)	0.929	0.929	1.01 (0.69–1.47)	0.979	0.979	1.07 (0.73–1.57)	0.715	0.715
		TT	0.68 (0.40–1.14)	0.143	0.428	0.88 (0.52–1.48)	0.622	0.622	1.03 (0.62–1.72)	0.901	0.901	0.57 (0.33–0.98)	**0.041**	0.245
		CC	1			1			1			1		
	Dominant	TC-TT	0.94 (0.66–1.36)	0.759	0.911	0.98 (0.69–1.40)	0.924	0.924	1.01 (0.71–1.45)	0.949	0.949	0.93 (0.65–1.34)	0.696	0.696
	Recessive	CC-TC	1			1			1			1		
		TT	0.66 (0.41–1.05)	0.078	0.465	0.87 (0.54–1.40)	0.560	0.560	1.03 (0.65–1.62)	0.900	0.928	0.54 (0.33–0.89)	**0.016**	1.977
	Log-additive	–	0.86 (0.67–1.11)	0.240	0.719	0.95 (0.74–1.23)	0.709	0.709	1.01 (0.79–1.30)	0.911	0.911	0.82 (0.63–1.05)	0.120	0.239

OR, odds ratio, 95% CI; 95% confidence intervals; FDR, false discovery rate.

The *p*^a^-values were calculated by logistic regression analysis adjusted by age, gender, and drinking. The *p*^b^-values were calculated by logistic regression analysis adjusted by age, gender, and smoking.

A *p* < 0.05 indicates statistical significance.

Significant *p*-values are given in bold.

**Table 6:** The relationship between *CYP11B1* polymorphisms and the risk of ischemic stroke complicated with hypertension.

**SNP ID**	**Model**	**Allele/Genotype**	**Case N**	**Control N**	**OR (95% CI)**	***p***	***p* (FDR)**
rs4736312	Allele	C	658	911	1		
		A	112	187	0.83 (0.64–1.07)	0.149	0.299
	Codominant	AC	100	159	0.84 (0.62–1.12)	0.235	0.469
		AA	6	14	0.59 (0.22–1.55)	0.283	0.566
		CC	279	376	1		
	Dominant	AC-AA	106	173	0.82 (0.61–1.09)	0.167	0.334
	Recessive	CC-AC	379	535	1		
		AA	6	14	0.62 (0.23–1.63)	0.330	0.672
	Log-additive	–	–	–	0.82 (0.63–1.06)	0.129	0.259
rs5017238	Allele	A	654	912	1		
		G	114	188	0.85 (0.66–1.09)	0.194	0.291
	Codominant	AG	98	156	0.84 (0.63–1.14)	0.263	0.394
		GG	8	16	0.68 (0.29–1.62)	0.388	0.466
		AA	278	378	1		
	Dominant	AG-GG	106	172	0.83 (0.62–1.11)	0.202	0.303
	Recessive	AA-AG	376	534	1		
		GG	8	16	0.72 (0.30–1.69)	0.448	0.580
	Log-additive	–	–	–	0.84 (0.65–1.08)	0.176	0.264
rs5301	Allele	C	653	912	1		
		T	117	188	0.87 (0.68–1.12)	0.275	0.330
	Codominant	TC	103	160	0.87 (0.65–1.16)	0.342	0.411
		TT	7	14	0.69 (0.27–1.74)	0.433	0.433
		CC	275	376	1		
	Dominant	TC-TT	110	174	0.85 (0.64–1.14)	0.278	0.333
	Recessive	CC-TC	378	536	1		
		TT	7	14	0.72 (0.29–1.81)	0.484	**0.009**
	Log-additive	–	–	–	0.86 (0.66–1.11)	0.241	0.289
rs5283	Allele	G	516	803	1		
		A	254	295	1.34 (1.10–1.64)	**0.004**	**0.026**
	Codominant	GA	168	225	1.24 (0.94–1.64)	0.122	0.733
		AA	43	35	2.07 (1.27–3.36)	**0.003**	**0.020**
		GG	174	289	1		
	Dominant	GA-AA	211	260	1.35 (1.04–1.76)	**0.024**	0.143
	Recessive	GG-GA	342	514	1		
		AA	43	35	1.87 (1.17–2.98)	**0.009**	0.233
	Log-additive	–	–	–	1.35 (1.10–1.66)	**0.004**	**0.022**
rs6410	Allele	C	568	761	1		
		T	202	339	0.80 (0.65–0.98)	**0.031**	0.094
	Codominant	TC	164	245	0.85 (0.65–1.11)	0.231	0.692
		TT	19	47	0.52 (0.29–0.91)	**0.022**	0.067
		CC	202	258	1		
	Dominant	TC-TT	183	292	0.79 (0.61–1.03)	0.085	0.255
	Recessive	CC-TC	366	503	1		
		TT	19	47	0.56 (0.32–0.97)	**0.039**	0.875
	Log-additive	–	–	–	0.78 (0.63–0.97)	**0.025**	0.074
rs4534	Allele	C	464	639	1		
		T	306	461	0.91 (0.76–1.10)	0.348	0.348
	Codominant	TC	192	269	0.96 (0.72–1.29)	0.804	0.804
		TT	57	96	0.81 (0.54–1.20)	0.290	0.434
		CC	136	185	1		
	Dominant	TC-TT	249	365	0.92 (0.70–1.21)	0.566	0.566
	Recessive	CC-TC	328	454	1		
		TT	57	96	0.82 (0.58–1.18)	0.292	0.599
	Log-additive	–	–	–	0.91 (0.75–1.10)	0.337	0.337

OR, odds ratio, 95% CI; 95% confidence intervals; FDR, false discovery rate.

The p-values were calculated by logistic regression analysis adjusted by age, gender, smoking, and drinking.

*p* < 0.05 indicates statistical significance.

### FRPR results

We performed the FPRP analysis to verify the positive data in the study. As shown in Supplementary Table S1, it was found that the associations between *CYP11B1* gene polymorphism and IS in the total group and subgroup, almost all of them, were significant (FPRP < 0.2).

### SNP–SNP interactions influenced ischemic stroke risk

**Figure 1:**
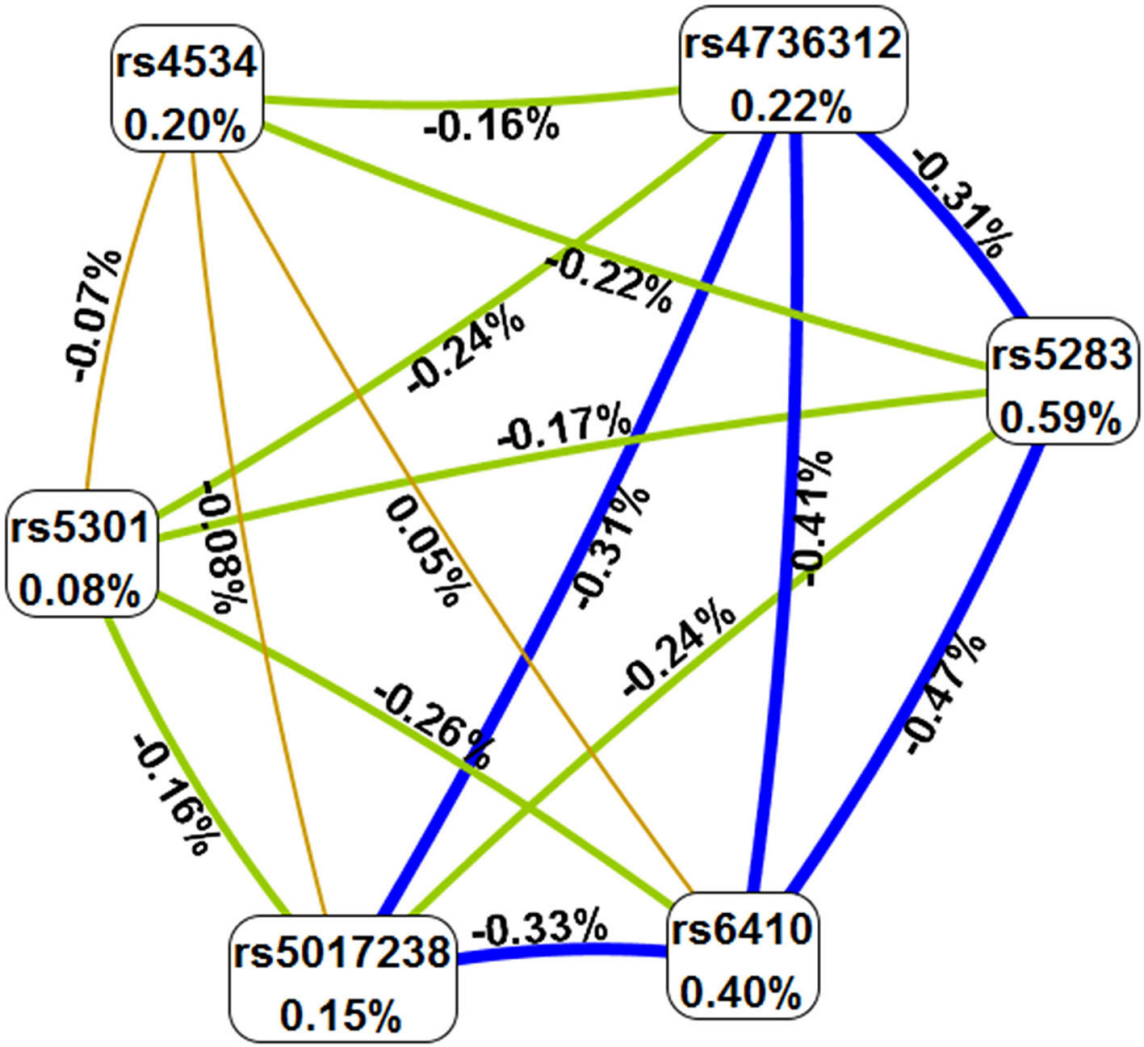
The SNP–SNP interaction map. Values in nodes represent the information gains of the individual attributes (main effects). Values between nodes are information gains of each pair of attributes (interaction effects). Orange with positive percent entropy indicates a strong synergistic interaction. Light range, blue, and green with negative percent entropy indicate redundancy or independence.

The MDR method was used to analyze the correlation between SNP–SNP interactions and IS. As presented in Table 7, rs5283 was the best predictive model for IS (OR 1.39, *p* = 0.007), with the highest testing accuracy (0.5400) and perfect cross-validation consistently (CVC) (10/10). The interaction map showed that rs4534 and rs6410 had a positive synergistic interaction (0.05%), and the interaction map with negative percent entropy indicated the redundancy or independence of each pairwise combination of SNPs (Figure 1).

**Table 7:** Best models to predict ischemic stroke by MDR.

**Model**	**Testing bal. acc**.	**CVC**	**OR (95% CI)**	***p***
rs5283	0.5400	10/10	1.39 (1.10–1.76)	**0.007**
rs6410, rs4534	0.5327	10/10	1.46 (1.15–1.85)	**0.002**
rs5017238, rs5301, rs5283	0.5036	5/10	1.51 (1.19–1.91)	**<0.001**
rs5017238, rs5283, rs6410, rs4534	0.4982	5/10	1.56 (1.23–1.99)	**<0.001**
rs5017238, rs5301, rs5283, rs6410, rs4534	0.5055	7/10	1.58 (1.24–2.01)	**<0.001**
rs4736312, rs5017238, rs5301, rs5283, rs6410, rs4534	0.5055	10/10	1.58 (1.24–2.01)	**<0.001**

The model with the maximum testing accuracy and maximum CVC was considered the best model.

The *p*-values were validated by 1,000 permutation tests. Significant *p*-values are given in bold.

Bal. Acc., balanced accuracy; CVC, cross-validation consistently; MDR, multifactor dimensionality reduction.

## Discussion

We studied the impact of *CYP11B1* SNPs on the IS risk. We found that rs5283 and rs6410 were closely related to the risk of IS. To the best of our knowledge, this study is the first of its kind to reveal an association between the *CYP11B1* gene polymorphisms and the risk of stroke in the Han population.

Rs5283, rs6410, rs4736312, and rs4534 are located on the second exon, the first exon, the 3′ UTR, and the first exon of the *CYP11B1* gene, respectively. Our study showed that rs5283 increases the risk of IS significantly, whereas rs6410 played a protective role in IS. However, Zhang et al. (2010) reported that rs6410 was associated with an increased risk of primary hyperaldosteronism. This difference may be caused by the type of disease. The incidence of stroke is proportional to age, with ~75% of stroke occurring in patients above 64 years (Mackay et al., 2004). The average age of the participants was 63 years; thus, we stratified the age group by 63 years. We found that rs5283 was associated with an increased susceptibility to IS in people aged >63 years. On the contrary, rs6410 decreased susceptibility to IS in people aged >63 years. Yang et al. (2020) reported that rs6068816 enhanced the IS risk in people aged >64 years. Cai et al. (2020) showed that rs4646 could increase the IS risk in people aged >64 years. Besides, rs2074633 and rs28688791 enhanced the risk of stroke in people aged <60 years (Wang et al., 2019). Taking various points from the above, we speculate that the association of *CYP11B1* gene polymorphisms with IS susceptibility may rely on age. In addition, we observed that rs5283 and rs6410 were closely related to IS risk in women. Some studies showed that SNPs were related to IS susceptibility but influenced by gender (Xu et al., 2017; Gu et al., 2018; Yuan et al., 2021). Besides, sex differences are very important to influence the occurrence of IS (Bushnell et al., 2018). Thus, we guess that *CYP11B1* genetic variants impact on the risk of IS relying on gender. Smoking and hypertension are risk factors for IS. We also analyzed the correlation between *CYP11B1* polymorphisms and IS risk stratified by smoking and hypertension. We observed that rs5283 could enhance the risk of IS in patients that do not smoke and those with hypertension. Rs6410 was related to decreased susceptibility to IS risk in patients that do not smoke and those with hypertension. In addition, rs473631 polymorphism reduced IS risk in patients that do not smoke. Similar to our results, Aysun et al. revealed that genetic variants determine IS risk influenced by hypertension and smoking (Türkanoglu Özçelik et al., 2018). Tu, Yang, and Diakite showed that SNPs were related to the susceptibility of IS with hypertension (Diakite et al., 2016; Tu et al., 2020; Yang et al., 2020). Cheng et al. (2017) showed that the interactions between SNPs and smoking turned out to be significant in IS. Based on the above, we concluded that gene polymorphisms together with age, gender, smoking, and hypertension are very significant risk factors for IS.

Function prediction found that rs5283 and rs4736312 were related to the regulation of deoxyribonuclease (DNAse), motifs changed, and selected expression quantitative trait loci (eQTL) hits. Rs6410 influences the regulation of promoter histone marks, enhancer histone marks, motifs changed, GRASP (Genome-Wide Repository of Associations Between SNPs and Phenotypes) QTL hits, and selected eQTL hits. Besides, rs4534 contributed to the regulation of promoter histone marks, DNAse, motifs changed, and enhancer histone marks, which have given that these SNPs had some molecular functions in IS. Studies showed that SNPs participate in the occurrence of human diseases by regulating the expression of the gene (Alvarez-Madrazo et al., 2013; Song et al., 2020). We estimate that *CYP11B1* gene polymorphism may affect the occurrence of the disease by regulating its expression, and molecular experiments have to be carried out to verify it.

There are a few disadvantages in our study. First, SNPs in *CYP11B1* may influence the occurrence of IS by regulating the expression of *CYP11B1*, but we have not detected a similar trait in the current study, so, this aspect merits future investigation. Second, the functional experiments of *CYP11B1* gene polymorphisms in patients with IS are still lacking in clarity and require further investigation and an in-depth study. Despite these limitations, our study is the first to explore the roles of *CYP11B1* gene polymorphisms in patients with IS.

## Conclusion

In summary, our study provided evidence that *CYP11B1* gene polymorphisms influence IS susceptibility in the Chinese Han population, which has given a new biomarker for the diagnosis and prevention of IS.

## Data availability statement

The original contributions presented in the study are publicly available. This data can be found here: https://doi.org/10.5281/zenodo.7344449.

## Ethics statement

The studies involving human participants were reviewed and approved by Xianyang Central Hospital. The patients/participants provided their written informed consent to participate in this study.

## Author contributions

YD conceived, designed the experiments, and revised the manuscript. GL performed the experiment, analyzed the data, and wrote the manuscript. Both authors read and approved the final manuscript.

## Conflict of interest

The authors declare that the research was conducted in the absence of any commercial or financial relationships that could be construed as a potential conflict of interest.

## Publisher's note

All claims expressed in this article are solely those of the authors and do not necessarily represent those of their affiliated organizations, or those of the publisher, the editors and the reviewers. Any product that may be evaluated in this article, or claim that may be made by its manufacturer, is not guaranteed or endorsed by the publisher.
