# Supplementary material for: CYP11B1 gene polymorphisms and susceptibility to ischemic stroke in a Chinese Han population
Source: Front Neurosci. 2022 Dec 1;16:1030551. doi: 10.3389/fnins.2022.1030551 (PMC9752905; doi:10.3389/fnins.2022.1030551)
Supplement: Supplementary file 1 [file Table_1.DOCX]

Table S1 False-positive report probability analysis for the positive findings between *CYP11B1* polymorphisms and ischemic stroke risk

| Genotype and Variables | OR (95 % CI) | *p* Value ^a^ | Statistical Power ^b^ | Prior Probability | | | |  |
| --- | --- | --- | --- | --- | --- | --- | --- | --- |
|  |  |  |  | 0.25 | 0.1 | 0.01 | 0.001 | 0.0001 |
| rs5283 G > A |  |  |  |  |  |  |  |  |
| A Vs G | 1.32 (1.10-1.58) | 0.003 | 1.000 | 0.007 ^c^ | 0.022 ^c^ | 0.197 ^c^ | 0.712 | 0.961 |
| GA Vs GG | 1.33 (1.04-1.70) | 0.026 | 0.999 | 0.064 ^c^ | 0.170 ^c^ | 0.693 | 0.958 | 0.996 |
| AA Vs GG | 1.81 (1.14-2.86) | 0.012 | 0.666 | 0.047 ^c^ | 0.130 ^c^ | 0.621 | 0.943 | 0.994 |
| GA-AA Vs GG | 1.39 (1.10-1.77) | 0.007 | 0.998 | 0.022 ^c^ | 0.064 ^c^ | 0.429 | 0.883 | 0.987 |
| AA Vs GG-GA | 1.58 (1.01-2.47) | 0.044 | 0.849 | 0.137 ^c^ | 0.322 | 0.839 | 0.981 | 0.998 |
| rs6410 C > T |  |  |  |  |  |  |  |  |
| T Vs C | 0.81 (0.67-0.98) | 0.027 | 1.000 | 0.083 ^c^ | 0.214 | 0.749 | 0.968 | 0.997 |
| TT Vs CC | 0.56 (0.34-0.91) | 0.020 | 0.676 | 0.079 ^c^ | 0.204 | 0.738 | 0.966 | 0.996 |
| TT Vs CC-TC | 0.60 (0.37-0.97) | 0.037 | 0.772 | 0.126 ^c^ | 0.302 | 0.827 | 0.980 | 0.998 |
| **Age> 63 years** |  |  |  |  |  |  |  |  |
| rs5283 G > A |  |  |  |  |  |  |  |  |
| A Vs G | 1.38 (1.07-1.79) | 0.015 | 0.997 | 0.044 ^c^ | 0.121 ^c^ | 0.602 | 0.938 | 0.993 |
| GA Vs GG | 1.58 (1.08-2.30) | 0.018 | 0.891 | 0.054 ^c^ | 0.146 ^c^ | 0.653 | 0.950 | 0.995 |
| AA Vs GG | 2.41 (1.23-4.73) | 0.011 | 0.294 | 0.097 ^c^ | 0.244 | 0.781 | 0.973 | 0.997 |
| GA-AA Vs GG | 1.69 (1.18-2.42) | 0.005 | 0.821 | 0.015 ^c^ | 0.044 ^c^ | 0.335 | 0.836 | 0.981 |
| AA Vs GG-GA | 1.93 (1.01-3.68) | 0.047 | 0.543 | 0.202 | 0.432 | 0.893 | 0.988 | 0.999 |
| rs6410 C > T |  |  |  |  |  |  |  |  |
| TT Vs CC | 0.43 (0.20-0.93) | 0.032 | 0.351 | 0.215 | 0.451 | 0.900 | 0.989 | 0.999 |
| **Women** |  |  |  |  |  |  |  |  |
| rs5283 G > A |  |  |  |  |  |  |  |  |
| A Vs G | 1.87 (1.40-2.52) | < 0.001 | 0.671 | < 0.001 ^c^ | 0.001 ^c^ | 0.006 ^c^ | 0.055 ^c^ | 0.369 |
| GA Vs GG | 2.24 (1.48-3.37) | < 0.001 | 0.293 | 0.001 ^c^ | 0.003 ^c^ | 0.035 ^c^ | 0.270 | 0.788 |
| AA Vs GG | 3.31 (1.58-6.90) | 0.001 | 0.089 | 0.045 ^c^ | 0.124 ^c^ | 0.609 | 0.940 | 0.994 |
| GA-AA Vs GG | 2.39 (1.61-3.53) | < 0.001 | 0.185 | < 0.001 ^c^ | 0.001 ^c^ | 0.006 ^c^ | 0.060 ^c^ | 0.392 |
| AA Vs GG-GA | 2.25 (1.11-4.56) | 0.024 | 0.372 | 0.165 ^c^ | 0.372 | 0.867 | 0.985 | 0.998 |
| rs6410 C > T |  |  |  |  |  |  |  |  |
| T Vs C | 0.64 (0.47-0.86) | 0.003 | 0.949 | 0.010 ^c^ | 0.028 ^c^ | 0.243 | 0.764 | 0.970 |
| TC Vs CC | 0.67 (0.45-0.99) | 0.045 | 0.929 | 0.125 ^c^ | 0.301 | 0.825 | 0.979 | 0.998 |
| TT Vs CC | 0.30 (0.13-0.70) | 0.006 | 0.119 | 0.119 ^c^ | 0.289 | 0.817 | 0.978 | 0.998 |
| TC-TT Vs CC | 0.60 (0.41-0.88) | 0.009 | 0.825 | 0.032 ^c^ | 0.089 ^c^ | 0.518 | 0.916 | 0.991 |
| TT Vs CC-TC | 0.36 (0.16-0.83) | 0.017 | 0.220 | 0.184 ^c^ | 0.403 | 0.881 | 0.987 | 0.999 |
| **Non-smoking** |  |  |  |  |  |  |  |  |
| rs4736312 C > A |  |  |  |  |  |  |  |  |
| A Vs C | 0.72 (0.52-0.99) | 0.045 | 0.988 | 0.116 ^c^ | 0.282 | 0.812 | 0.978 | 0.998 |
| rs5283 G > A |  |  |  |  |  |  |  |  |
| A Vs G | 1.40 (1.08-1.81) | 0.012 | 0.997 | 0.030 ^c^ | 0.085 ^c^ | 0.504 | 0.911 | 0.990 |
| GA Vs GG | 1.62 (1.13-2.32) | 0.008 | 0.875 | 0.028 ^c^ | 0.080 ^c^ | 0.489 | 0.906 | 0.990 |
| AG-AA Vs GG | 1.64 (1.16-2.32) | 0.005 | 0.869 | 0.018 ^c^ | 0.051 ^c^ | 0.371 | 0.856 | 0.984 |
| rs6410 C > T |  |  |  |  |  |  |  |  |
| T Vs C | 0.74 (0.57-0.97) | 0.027 | 0.998 | 0.081 ^c^ | 0.209 | 0.744 | 0.967 | 0.997 |
| TT Vs CC | 0.42 (0.21-0.86) | 0.017 | 0.317 | 0.143 ^c^ | 0.334 | 0.847 | 0.982 | 0.998 |
| TT Vs CC-TC | 0.46 (0.23-0.92) | 0.029 | 0.407 | 0.172 ^c^ | 0.383 | 0.872 | 0.986 | 0.999 |
| **Non-drinking** |  |  |  |  |  |  |  |  |
| rs4534 C > T |  |  |  |  |  |  |  |  |
| TT Vs CC | 0.57 (0.33-0.98) | 0.041 | 0.682 | 0.156 ^c^ | 0.357 | 0.859 | 0.984 | 0.998 |
| TT Vs CC-TC | 0.54 (0.33-0.89) | 0.016 | 0.619 | 0.071 ^c^ | 0.185 ^c^ | 0.715 | 0.962 | 0.996 |
| **Hypertension** |  |  |  |  |  |  |  |  |
| rs5283 G > A |  |  |  |  |  |  |  |  |
| A Vs G | 1.34 (1.10-1.64) | 0.004 | 1.000 | 0.013 ^c^ | 0.039 ^c^ | 0.309 | 0.819 | 0.978 |
| AA Vs GG | 2.07 (1.27-3.36) | 0.003 | 0.445 | 0.021 ^c^ | 0.062 ^c^ | 0.419 | 0.879 | 0.986 |
| GA-AA Vs GG | 1.35 (1.04-1.76) | 0.024 | 0.998 | 0.074 ^c^ | 0.193 ^c^ | 0.725 | 0.964 | 0.996 |
| AA Vs GG-GA | 1.87 (1.17-2.98) | 0.009 | 0.611 | 0.040 ^c^ | 0.111 ^c^ | 0.578 | 0.933 | 0.993 |
| rs6410 C > T |  |  |  |  |  |  |  |  |
| TVs C | 0.80 (0.65-0.98) | 0.031 | 1.000 | 0.085 ^c^ | 0.219 | 0.755 | 0.969 | 0.997 |
| TT Vs CC | 0.52 (0.29-0.91) | 0.022 | 0.555 | 0.106 ^c^ | 0.263 | 0.797 | 0.975 | 0.997 |
| TT Vs CC-TC | 0.56 (0.32-0.97) | 0.039 | 0.657 | 0.150 ^c^ | 0.346 | 0.853 | 0.983 | 0.998 |

*p* value ^a^ was calculated by unconditional logistic regression analysis with adjustment for age and gender.

Statistical power ^b^ was calculated using the number of observations in the subgroup and the OR and *p* values in this table.

^c^ The level of false-positive report probability threshold was set at 0.2 and noteworthy findings are presented.
